# Enhancing Early Drought Detection in Plants: The Consideration of Organ Sensitivity, Parameter Selection, and Measurement Timing

**DOI:** 10.3390/plants14111571

**Published:** 2025-05-22

**Authors:** Guanqiang Zuo, Naijie Feng, Dianfeng Zheng

**Affiliations:** 1 College of Coastal Agricultural Sciences, Guangdong Ocean University, Zhanjiang 524008, China; byndzgq@163.com (G.Z.);; 2 College of Natural Resources and Environment, Northwest A&F University, Yangling 712100, China

**Keywords:** drought, organ sensitivity, parameter sensitivity, time sensitivity, photosynthesis, legumes

## Abstract

Drought stress constitutes one of the most severe constraints to global agricultural productivity. Early drought detection is pivotal for sustainable agriculture, yet current approaches overlook critical dimensions of plant sensitivity. While advancements in photosynthetic parameter analysis (e.g., gas exchange, and chlorophyll fluorescence) have enhanced drought monitoring, three understudied factors limit progress: (1) differential drought sensitivity across plant organs (e.g., root nodules vs. leaves); (2) the selection of sensitive photosynthetic parameters and optimal measurement timing for stress detection; and (3) the identification of leaf layers most responsive to water deficits. By synthesizing insights from nodule physiology in legumes, cross-species evidence on multi-layered leaf senescence, and the temporal dynamics of stress sensitivity, this paper proposes a ‘whole-plant sensitivity analysis’ framework. Integrating organ-, parameter-, and time-specific perspectives, this paper aims to refine early drought detection in the field and enhance plant resilience research.

## 1. Introduction

Drought, often referred to as a “creeping disaster”, is a slow onset yet highly destructive stressor that is being increasingly intensified by global climate change. Current estimates suggest that drought threatens more than 50% of global croplands [1]. Modern agricultural practices exacerbate this crisis. For example, high-density maize planting while advantageous for yield maximization under optimal conditions increases vulnerability to water scarcity by intensifying root-zone competition and reducing the soil’s buffering capacity for drought events [2]. Alarmingly, recent studies indicated that the rate of drought intensification is accelerating, posing an even greater threat to global food security [3]. This trend underscores the urgent need for effective early warning systems to facilitate timely management interventions and mitigate the adverse impacts of drought on agricultural productivity and yield stability.

In addition, a fundamental question in this context is whether the current improvements in photosynthetic efficiency (improving the conversion efficiency of intercepted radiation into biomass [4]), driven by breeding efforts to optimize crop yields under non-stressed conditions, have inadvertently increased crops’ tolerance to drought. Some argue that while selecting for high yield potential may offer some benefits, it is not a direct strategy for improving drought resilience [5]. Certain high-yielding lines, selected under optimal conditions, have shown increased yield under drought stress, potentially due to spillover effects from selection for other traits [6]. This suggests that improved yield potential does not necessarily equate to enhanced drought resilience, emphasizing the critical role of drought management strategies, both in breeding programs and broader agricultural practices.

Since the late 20th century, scientific research on drought in agriculture has experienced exponential growth, driven by the global threat of water scarcity under climate change scenarios [7]. This research surge reflects both the scientific community’s recognition of drought as a major constraint to agricultural productivity and the urgent demand for adaptive strategies to secure food production in water-limited environments. At the physiological level, drought stress fundamentally alters plant–water relations, with predawn leaf water potential (Ψpd), measured using a pressure chamber, serving as a sensitive and early indicator of declining soil moisture availability [8]. A drop in Ψpd often precedes visible wilting and functions as a primary signal that initiates a cascade of downstream stress responses. In addition to Ψpd, midday leaf water potential (Ψmd) also provides valuable insights into plant water balance during peak evaporative demand. The combined use of Ψpd and Ψmd allows researchers to differentiate between soil-driven and atmospheric components of drought stress and is particularly useful in screening for genotypic differences in drought response.

Among the downstream processes affected by drought, photosynthesis—the primary driver of biomass accumulation—is especially sensitive. Water stress leads to a progressive series of impairments, including stomatal closure, restricted CO_2_ diffusion, reduced mesophyll conductance, and photoinhibition. It also disrupts the delicate balance between photochemical and non-photochemical energy dissipation pathways. As such, photosynthetic traits represent a valuable window into the early physiological responses of crops under drought stress and form the basis for many modern diagnostic approaches.

At the regional scale, advances in remote sensing have enabled early drought detection through metrics such as sun-induced chlorophyll fluorescence yield (SIF_yield_), which can reveal drought stress approximately one month earlier than traditional vegetation indices or gross SIF signals [9]. On the plant level, however, much of the research has focused on understanding how drought affects growth, photosynthesis, and chlorophyll fluorescence [10,11,12]. While several studies have explored various parameters and indicators for early drought detection [13,14], these efforts remain fragmented and have not yet resulted in comprehensive or universally applicable solutions.

Recent advances in high-resolution physiological monitoring offer unprecedented opportunities to probe drought responses at the whole-plant level, especially through photosynthetic diagnostics. While foundational studies have established robust theoretical frameworks and standardized protocols in photosynthesis determination [15,16,17], three critical knowledge gaps persist in early drought diagnostics:

1. Organ sensitivity. Current drought phenotyping methodologies remain disproportionately focused on foliar responses, creating a critical blind spot in our understanding of whole-plant stress adaptation. This leaf-centric approach potentially overlooks more sensitive and biologically significant stress indicators in other organs, such as roots and nodules. Therefore, this fundamental consideration of organ-level response variation must be addressed at the experimental design stage to ensure comprehensive stress detection.
2. Parameter prioritization. While modern tools (e.g., chlorophyll fluorescence coupled gas exchange systems) provide numerous photosynthetic parameters, their relative sensitivity as early drought indicators remain unclear. Rigorous validation is needed to identify the most reliable biomarkers for pre-visual stress detection. This evaluation should specifically examine which parameters in which leaf layers show the earliest and most consistent responses to drought stress.
3. Temporal specificity. Drought responses are not static—they fluctuate diurnally with light intensity, temperature, and vapor pressure deficit. As a result, the time of day at which physiological measurements are taken can significantly influence their diagnostic value. Identifying optimal measurement windows and understanding how measurement timing affects detection sensitivity are key to improving the reliability of early warning systems.

Legumes, the third-largest family of flowering plants, serve as a primary source of dietary protein and oil while playing a pivotal role in sustainable agriculture through symbiotic nitrogen fixation [18]. The formation of root nodules allows legumes to convert atmospheric nitrogen into bioavailable forms, reducing dependence on synthetic fertilizers and lowering the environmental footprint of crop production [19]. However, legumes exhibit less efficient photosynthetic biochemistry than cereals [20], and greater drought sensitivity [20].

Despite these limitations, legumes offer a unique and underutilized model system for advancing early drought detection strategies. Their distinctive nodule–root–shoot architecture provides a natural framework to investigate organ-specific stress responses. While previous studies suggest that legume leaves are more sensitive to drought than roots [21], the comparison of sensitivity between nodules and leaves to drought remains an open question. By synthesizing evidence from legume-focused studies (organ sensitivity) and cross-species reports (parameter/time sensitivity), we advocate a ‘systems-level approach’ for early drought detection. Such an approach not only enhances our understanding of plant stress physiology but also holds promise for improving yield in both legumes and non-leguminous crops through informed management strategies.

## 2. Drought Sentinels Within Plants: Nodules or Leaves

**Figure 1:**
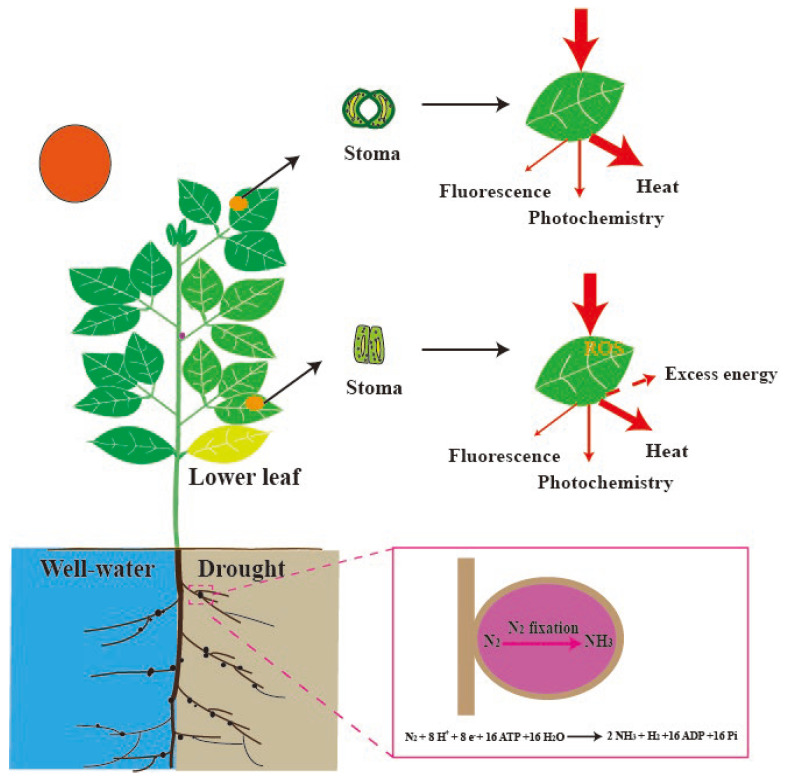
Early Drought Detection in Soybeans. In soybeans, nitrogen fixation—a key physiological process mediated by symbiotic root nodules—is highly sensitive to soil water deficit. Under drought conditions, nitrogenase activity within the nodules declines significantly, often serving as one of the earliest physiological indicators of stress. Simultaneously, a vertical gradient in drought sensitivity is observed within the canopy, with lower leaves exhibiting greater vulnerability compared to upper leaves. These lower leaves tend to undergo stomatal closure earlier in response to declining leaf water potential, leading to restricted CO_2_ uptake. Despite this reduction in gas exchange, they continue to intercept incident light—particularly during transient high light events caused by canopy gaps or sunflecks. This imbalance between energy absorption and utilization increases the risk of excess excitation pressure on photosystem II (PSII), making these leaves more prone to photoinhibition and oxidative damage. Together, the decline in nodule nitrogenase activity and the increased photosynthetic vulnerability of lower leaves highlight their potential utility as sensitive physiological markers for early drought detection in soybean. ROS, reactive oxygen species.

Leguminous plants form symbiotic associations with nitrogen-fixing rhizobia, leading to the development of root nodules (Figure 1). The development of nodules involves the tightly regulated coordination of two parallel but interconnected programs: (1) the infection of root hairs at the epidermal level, where rhizobia gain entry through infection threads, and (2) nodule organogenesis in the root cortex, where the nodule primordium develops from dedifferentiated cortical cells [22,23]. These processes are regulated by complex hormonal signaling networks, including auxins, cytokinins, gibberellins, and abscisic acid (ABA), which, together, ensure spatial and temporal synchronization between rhizobial invasion and nodule formation [23]. Importantly, ABA also plays a central role in plant drought responses, where it regulates key physiological processes such as stomatal closure [24]. Emerging evidence reveals that root nodules are extremely sensitive to ABA, even at low concentrations. For instance, exposure to as little as 0.5 µM ABA has been shown to significantly inhibit nodulation, while exerting minimal effects on overall root or shoot growth [25]. This ABA-induced suppression of nodulation has been observed across various legume species, including *Lotus japonicus* [26,27], *Medicago truncatula* [28], and *Trifolium repense* [29]. These findings suggest that the inhibitory effect of ABA on nodulation may represent a conserved regulatory mechanism that is particularly critical during early-stage drought stress, when plants must prioritize survival over resource-intensive symbiotic processes.

Direct experimental evidence demonstrates that nitrogenase activity in soybean nodules—typically quantified using the acetylene reduction assay (ARA)—exhibits significantly higher sensitivity to soil water deficits than leaf-level gas exchange parameters. This observation strongly supports the hypothesis that biological nitrogen fixation (BNF), and by extension nodulation processes, can serve as early and reliable physiological indicators of drought stress [30]. In an earlier study, Albrecht et al. [31] provided compelling comparative data, showing that within just four days of water deprivation, nitrogenase activity declined precipitously by approximately 70%, whereas the net photosynthetic rate exhibited only a modest 5% reduction over the same period. These findings suggested that drought-induced inhibition of nitrogenase activity is not merely a downstream consequence of reduced photosynthesis or carbohydrate availability, but, rather, may involve direct physiological or biochemical disruption of the nodule environment. Recent advances have further revealed that legumes with determinate nodules show greater drought vulnerability than indeterminate-nodule species [32], suggesting nodule types also impact stress resilience.

However, the “nodule-first” hypothesis faces challenge. A previous study indicated that the lower leaves typically initiate drought-induced senescence, reallocating resources to the upper leaves and nodules [33]. This pattern of resource redistribution is considered an evolutionary strategy to enhance drought resilience, allowing the plant to prioritize the survival and functionality of photosynthetically efficient tissues [33]. While sequential leaf senescence is a common feature in higher plants [34], most research on photosynthetic responses to drought has focused on upper leaves, typically the first or second fully expanded leaves while largely neglecting the middle and lower layers. Cai et al. [35] reported that drought stress caused significant reductions in the net photosynthetic rate (Pn) across all maize leaf layers except the uppermost, suggesting that lower and middle leaves may be more vulnerable to water deficit. A few additional studies have explored leaf-layer-specific responses to drought stress [36,37], yet such integrative approaches remain rare. Given these, we should identify the most drought-sensitive leaf layers, with particular attention to lower leaves that may serve as early stress indicators. Implementing such assessments would not only advance our understanding of plant adaptation mechanisms but also provide novel insights into the early drought detection protocols.

## 3. Parameter Selection

Photosynthesis is highly sensitive to drought, with stomatal closures serving as one of the earliest physiological responses to water stress in field conditions [38,39,40]. This conservative water-saving strategy effectively reduces transpirational water loss, thus preserving plant water status. However, this response is a double-edged sword: although it reduces water loss, it also limits CO_2_ influx, potentially constraining carbon assimilation. Interestingly, studies have shown that Pn and electron transport rate (ETR) often remain relatively stable in the early stages of drought, provided that stomatal conductance (gs) does not drop below a critical threshold [12]. Drought-induced changes in gas exchange often led to an increase in intrinsic water use efficiency (WUEi, calculated as the ratio of Pn to gs). This enhancement of WUEi reflects the plant’s effort to maximize carbon gain per unit water loss, and it is particularly pronounced in C4 species. Due to their CO_2_-concentrating mechanism and naturally lower gs, C4 plants typically exhibit higher WUEi than C3 plants under both well-watered and drought conditions [41]. This physiological advantage contributes to the greater drought tolerance and productivity of C4 crops like maize and sorghum in arid and semi-arid environments.

At the molecular and biochemical level, stomatal behavior is primarily regulated by ABA and the redox state of plastoquinone (1-qL) [42]. The chlorophyll fluorescence parameter qL, which quantifies the fraction of open PSII reaction centers based on the “lake model”, provides a sensitive indicator of the functional status of the photosynthetic apparatus. qL can be precisely determined using pulse-amplitude modulation (PAM) fluorometry, a widely employed technique that measures rapid changes in chlorophyll fluorescence under ambient light conditions [43]. PAM instruments determine qL by analyzing fluorescence responses to a modulated measuring beam and brief saturating pulses of actinic light. Specifically, qL is calculated from the steady-state fluorescence (Fs), the maximum fluorescence during a saturating pulse (Fm′), and the minimum fluorescence in the light-adapted state (Fo′), using the equation qL = [(Fm′ − Fs)/(Fm′ − Fo′)] × (Fo′/Fs). qL values range from 0 (all centers closed) to 1 (all centers open), allowing researchers to monitor the dynamic balance between photochemical activity and regulatory feedback under varying environmental conditions, including drought stress.

**Figure 2:**
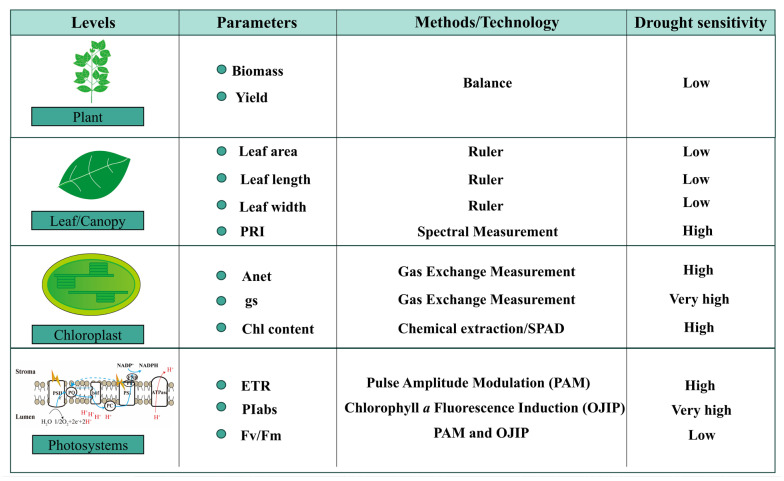
Sensitivity of different photosynthetic parameters across multiple levels. Photosynthetic responses to drought manifest at multiple hierarchical levels, from the molecular scale to whole-plant traits. This multi-level framework facilitates a comprehensive understanding of plant physiological adjustments under water deficit. At the photosystem level, key parameters such as the electron transport rate (ETR), photosynthetic performance index (PIabs), and maximum quantum efficiency of PSII photochemistry (Fv/Fm) provide early and sensitive indicators of photochemical function and stress-induced impairments in light energy conversion. At the chloroplast level, physiological metrics including net photosynthetic rate (Pn), stomatal conductance (gs), and chlorophyll content reflect changes in carbon assimilation capacity. At the leaf level, structural traits such as leaf length, leaf width, and leaf area are valuable indicators of morphological acclimation to drought, often reflecting cumulative effects on leaf expansion and development. The photochemical reflectance index (PRI), which captures xanthophyll cycle activity and light use efficiency, serves as a cross-scale parameter, applicable at both leaf and canopy levels. At the whole-plant level, biomass accumulation and grain yield represent the integrative outcomes of drought impact. These are ultimate indicators of drought sensitivity and crop performance, influenced by both photosynthetic efficiency and carbon allocation.

Notably, the response of the photosynthetic apparatus, particularly photosystem II (PSII), has been shown to be highly sensitive to a wide range of environmental stressors, including drought and high light intensity [44]. While chlorophyll content and morphology remain stable during early drought, fluorescence emission dynamics provide pre-visual stress signatures (Figure 2) [9]. In fact, chlorophyll fluorescence serves as a powerful, non-invasive probe of photosynthetic function. Upon absorption of light energy by chlorophyll molecules in the light-harvesting complexes, the excitation energy can follow one of three mutually exclusive pathways: (i) it can drive photochemical reactions in PSII (quantified as the photochemical yield, ΦP); (ii) it can be dissipated as heat via non-photochemical quenching processes (ΦN); or (iii) it can be re-emitted as fluorescence (ΦF). These three pathways are linked by the fundamental energy balance equation ΦP + ΦN + ΦF = 1 [15]. This principle allows researchers to derive meaningful insights into the efficiency and regulation of the photosynthetic light reactions by analyzing fluorescence parameters. For example, a decline in ΦP often reflects reduced photochemical efficiency, while a concurrent increase in ΦN may indicate the activation of photoprotective mechanisms such as NPQ to prevent excess excitation energy from damaging the photosynthetic machinery.

In addition, chlorophyll fluorescence-based metrics display a spectrum of sensitivities to drought stress, enabling nuanced detection of photosynthetic impairment across stress gradients. Among these, the maximum quantum efficiency of PSII photochemistry (Fv/Fm) has traditionally served as a key indicator of photoinhibition and damage to the PSII reaction center. However, Fv/Fm is relatively stable under mild to moderate drought conditions, with significant declines typically observed only when plants experience prolonged or severe water deficit [13,45]. In contrast, the photosynthetic performance index (PIabs), derived from chlorophyll a fluorescence induction (OJIP), responds much earlier, showing noticeable changes even at the initial stages of dehydration [46,47]. The enhanced sensitivity of PIabs stems from its composite nature, as it integrates multiple dimensions of PSII functionality. Specifically, it incorporates three core parameters from the JIP-test: (1) the density of active reaction centers (RC/ABS), (2) the quantum yield of primary photochemistry (φPo), and (3) the efficiency of electron transport beyond Q_A_ (ψEo). It should be noted that the OJIP curve is obtained within one second of saturating light exposure following dark adaptation (20–30 min). This rapid measurement reveals the stepwise reduction of electron acceptors through four characteristic phases: O (origin, Fo, ~50 µs), J (~2 ms), I (~30 ms), and P (peak fluorescence, Fm). The shape and amplitude of these phases provide a rapid, non-invasive snapshot of PSII performance and stress-induced disruptions. Taken together, the OJIP transient and derived JIP-test parameters, particularly PIabs, represent a technically robust and physiologically informative approach for capturing the early functional shifts in PSII photochemistry under drought.

Furthermore, parameters typically considered insensitive to mild drought can become highly responsive when combined with other environmental factors. This is because overlapping stresses create a “new state of environmental stress” distinct from isolated conditions [48]. This principle was effectively applied in Burke’s [49] innovative fluorescence-based drought detection method using Fv/Fm. The approach is based on the idea that drought-induced carbohydrate accumulation provides enhanced respiratory substrates. In detail, source leaf tissues from well-watered and water-stressed cotton plants were harvested at sunrise and exposed to prolonged respiratory demands (elevated temperature). In their study, the well-watered cotton exhibited a rapid decline in Fv/Fm, whereas the drought-stressed plants maintained higher Fv/Fm values. This methodology successfully amplifies subtle metabolic differences into detectable signals by leveraging the interaction between water deficit and heat stress, thereby establishing Fv/Fm as a sensitive early indicator of drought through stress potentiation.

In addition to gas exchange and fluorescence parameters, the photochemical reflectance index (PRI) has emerged as a valuable spectral indicator of photosynthetic efficiency, particularly under stress conditions. PRI is calculated using the narrowband reflectance at 531 nm and 570 nm, following the formula PRI = (R531 − R570)/(R531 + R570) [50,51]. This index is sensitive to the epoxidation state of xanthophyll pigments and has proven useful in capturing rapid changes in photosynthetic light-use efficiency across both leaf and canopy scales. Under conditions of high light intensity, especially when combined with drought stress, plants activate a key photoprotective mechanism—xanthophyll cycle-dependent NPQ. This mechanism serves to dissipate excess absorbed light energy as heat, thereby preventing photooxidative damage to PSII. The process involves the enzymatic de-epoxidation of violaxanthin to antheraxanthin, and zeaxanthin, pigments that play a direct role in facilitating energy dissipation within the light-harvesting complex. This pigment transformation is accompanied by a measurable shift in leaf reflectance, particularly in the green-yellow region near 531 nm, which forms the spectral foundation of PRI. Thénot et al. [52] demonstrated that PRI correlates strongly with plant water status and can serve as a non-invasive proxy for detecting early water stress. However, the reliability of PRI as a drought stress indicator is not absolute. Sarlikioti et al. [53] emphasized that PRI values are influenced by ambient light conditions.

## 4. Temporal Specificity: Diurnal Diagnostic Windows

Emerging evidence highlights that photosynthetic parameters exhibit circadian-modulated stress sensitivity, presenting a pivotal yet understudied question: does the timing of photosynthetic measurements fundamentally alter the detection threshold for early drought responses? Previously, Inamullah and Isoda [54] demonstrated time-dependent diagnostic precision in soybean and cotton under varying irrigation regimes. Midday/afternoon Pn measurements effectively distinguished between control and 50% irrigation treatments in soybean, while morning measurements showed no differences. Chen et al. [55] extended these observations to photochemical efficiency, reporting that the divergence in the effective quantum yield of PSII photochemistry (Φ_PSII_) between drought and control maize plants peaked at 12:00–16:00 local time. At the molecular level, Greenham et al. [56] attributed these diurnal variations in stress responses to differential rhythmic expression profiles of key stress-responsive genes and proteins, which are tightly coupled to the plant’s internal circadian clock. Despite these insights, significant knowledge gaps remain. While numerous studies have documented diurnal photosynthesis variation, few have systematically evaluated its utility for early drought detection. The potential synergy between diurnal timing and stress interaction effects (like those shown by Burke [49]) remains not well explored. Current research remains limited by narrow species representation and parameter selection, often focusing on single growth stages or photosynthetic components. A systematic investigation—spanning diverse crop species, developmental stages, and photosynthetic parameters—is urgently needed.

## 5. Concluding Remarks and Future Perspectives

A paradigm shift is needed to transform early drought detection from parameter-centric approaches to whole-plant systems analysis. This discussion establishes a sensitivity framework integrating organ, parameter, and measurement timing.

Innovative technologies have significantly advanced our understanding of crop physiology and yield improvement. However, these advancements also present new challenges, particularly in improving the specificity of drought stress monitoring. Environmental stresses often occur as compound events, involving multiple stressors such as pests, pathogens, drought, salinity, light, and heat, all of which influence stomatal conductance and PSII function. While molecular biology techniques, such as genetically encoded calcium reporters, have been used to monitor stress responses [57], these methods are not entirely specific to drought stress. Addressing this challenge requires collaborative efforts from diverse research groups.

For practical recommendations for photosynthetic monitoring under drought, we suggest a whole-canopy approach, i.e., determining representative leaves from different canopy layers. Lower canopy leaves, often shaded and metabolically older, can act as early stress indicators, showing symptoms such as chlorophyll degradation, PSII inactivation, and reduced photochemical efficiency before the upper leaves. Conversely, upper canopy leaves, which are more exposed to light, may exhibit compensatory mechanisms during early drought stress, such as increased photosynthetic electron transport and enhanced chlorophyll content [58,59]. Hence, multi-layered leaf analysis combined with sensitive photosynthetic indicators such as PIabs, Φ_PSII_, and PRI offers a robust, systems-level view of plant drought responses.

In addition to photosynthetic traits, osmotic adjustment plays a pivotal role in drought resilience. Plants maintain turgor pressure under water deficit by accumulating osmolytes such as proline, which exhibits strong drought-induced accumulation [60], and glycine betaine, a compatible solute that stabilizes enzymes, protein complexes, and membrane integrity [61]. Moving forward, research should focus on developing integrated, multi-parameter drought diagnostics that combine these key biochemical markers with photosynthetic performance data. Such integrative approaches will enable a more comprehensive and mechanistically informed understanding of plant responses to drought stress.

Moreover, future work should also focus on bridging the gap between laboratory-based precision measurements and field-scale applicability through the adoption of portable, high-throughput phenotyping platforms. Advanced tools such as drone-mounted multispectral and thermal imaging systems, ground-based SIF, and machine learning assisted image analysis algorithms provide robust, non-invasive capabilities for real-time monitoring of drought-induced physiological alterations across spatial and temporal scales. These platforms enable the quantitative assessment of key traits including photosynthetic efficiency, stomatal conductance, chlorophyll content, and canopy structural dynamics, thereby facilitating the early detection of stress responses prior to the onset of visible damage.

## Data Availability

No data was used for the research described in the article.
